# Global academic output on COVID-19 and Guillain-Barre Syndrome: A bibliometric analysis

**DOI:** 10.1016/j.heliyon.2022.e11354

**Published:** 2022-11-01

**Authors:** Carlos Quispe-Vicuña, Miguel Cabanillas-Lazo, Maria Eugenia Guerrero, Franco Mauricio, John Barja-Ore, Frank Mayta-Tovalino

**Affiliations:** a Sociedad Científica de San Fernando, Universidad Nacional Mayor de San Marcos, Lima, Peru; b Grupo Peruano de Investigación Epidemiológica, Unidad para la Generación y Síntesis de Evidencias en Salud, Universidad San Ignacio de Loyola, Lima, Peru; c Academic Department of Medical and Surgical Stomatology, Universidad Nacional Mayor de San Marcos, Lima, Peru; d Posgraduate Department, Universidad Nacional Federico Villarreal, Lima, Peru; e Dirección de Investigación, Universidad Continental, Huancayo, Peru; f Vicerrectorado de Investigación, Universidad San Ignacio de Loyola, Lima, Peru

**Keywords:** COVID-19, Guillain-Barre syndrome, Bibliometrics

## Abstract

The purpose of this study was to bibliometrically analyze scientific publications on Guillain-Barré syndrome (GBS) related to COVID-19. A specialized search of the Scopus was used (December 2019 to February 2022). Collected publications were evaluated in Scival (Elsevier). The results were arranged in tables for presentation. We found 959 papers that were collected and the highest percentage of these belonged to the area of Neurology. Josef Finsterer was the author with the highest academic production, but Benedict Michael was the one with the highest impact worldwide. Although the Universidade Federal de São Paulo (Brazil) was the college with the highest scientific production, it was King’s College London that reported the highest impact. Regarding the journals, the Journal of Neurology is the one with the highest worldwide production. In addition, an increase in first quartile publication and articles with national collaboration was reported. Scholarly output on COVID-19 and GBS have been increasing. Although national collaboration has the highest proportion of manuscripts, it is the international type that reported a greater impact, this would show a great interest on the part of researchers from all over the world regarding this topic.

## 1 Introduction

Guillain-Barré syndrome (GBS) is an immune-mediated polyradiculoneuropathy whose etiology is usually associated with an infectious process [1, 2]. The clinical presentation of this disease is heterogeneous; although its classic presentation is a progressive ascending limb weakness associated with loss of reflexes, patients may present with completely different clinical features [3, 4]. GBS has a global annual incidence rate of 1–2 cases per 100,000 people and, in 2019, it affected more than 150,000 people worldwide, especially in the Asia Pacific and North American regions [5, 6]. Moreover, despite treatment, death or severe disability can occur in up to 18% of cases [3]. For all these reasons, it is necessary to have a better understanding of the associated factors and etiological agents that can cause this disease. Although the most frequent cause of GBS is infection by the bacterium Campylobacter jejuni [7], there have also been reports that it can be caused by other pathogens such as cytomegalovirus, Haemophilus influenzae, Epstein-Barr virus, Zika virus and more recently COVID-19 [7, 8].

COVID-19 has evidenced a worldwide impact, as it has generated millions of cases and deaths, in addition to affecting health systems worldwide [9, 10]. Although possible pathophysiological mechanisms have been established between COVID-19 and GBS, such as an immunomodulated inflammatory process or a neuroinvasive process through ACE2 receptors in neuronal cells [11, 12, 13, 14]; the relationship between both diseases is not completely established [7]. For this reason, an analysis of the scholarly output on COVID-19 and GBS is necessary to have a better understanding of the current landscape and to establish new research opportunities for the future.

Bibliometric studies mainly allow the analysis of the characteristics of scholarly output in each field of knowledge [15]. This type of studies has gained some relevance due to the continuous growth of science. For example, several bibliometric studies focused on GBS [16, 17, 18] have been reported, even associating it with another viral disease such as ZIKA [19, 20].

Thus, the aim of this bibliometric study was to perform an analysis of the worldwide academic production related to COVID-19 and Guillain-Barré syndrome (2020–2022).

## 2 Materials and methods

### 2.1 Search strategy

The present study was a descriptive, bibliometric, and bibliometric design. We included original manuscripts related to COVID-19 and GBS (December 2019 and February 2022). Letters to the editor, proceedings and literature reviews were excluded.

The search was performed on February 15, 2022, using controlled language MeSH of PubMed to “COVID-19” and “Guillain-Barre Syndrome”. Also, Emtree terms from EMBASE were used to establish the search strategy. The following formula was used in Scopus:

TITLE-ABS-KEY (2019∗cov OR ncov OR (((cov) W/2 (19 OR 2019 OR 2)) AND NOT (“Coefficient∗ of variation” OR “Torsion” OR cov∗o∗)) OR (covid W/2 (19 OR 2019 OR 2)) OR covid19 OR (covid AND NOT tocovid) OR ((coronavirus OR “Corona virus” OR cov) W/2 (disease OR infection) W/2 (2019 OR 19 OR 2)) OR ((sars OR “Severe acute respiratory syndrome” OR SARS) W/2 (cov OR coronavirus OR “Corona virus” OR covid) W/2 (“2” OR 2019 OR 19)) OR “SARS-CoV2” OR sarscov2 OR “SARS-CoV2” OR “Severe acute respiratory syndrome COV2” OR ((((novel OR wuhan OR china OR pandemi OR outbreak OR “new human” OR crisis OR “new cases” OR “normalcy”) W/2 (coronaviru∗ OR “corona viru∗” OR covid)) OR (“new corona∗” AND NOT (coronar∗)))) OR “Corona pandemic” OR (wuhan W/2 pneumonia) OR “Corona crisis” OR “Corona outbreak” OR “20I 501Y.V1” OR “20J501Y.V3” OR “CAL.20C” OR “20H501Y.V2” OR “mRNA 1273 vaccine” OR “Covishield” OR “AZD1222” OR “Ad26.COV2.S” OR “JNJ 78436735” OR “Ad26COVS” OR “BNT162 vaccine” OR “BNT162-01” OR “BNT162b1” OR “BNT162a1” OR “BNT162b2” OR “BNT162c2”) AND TITLE-ABS-KEY (guillain∗ OR gbs OR “Acute neuropath∗” OR “Acute polyneuritis” OR “Miller Fisher” OR “Miller-Fisher” OR “Fisher Syndr∗” OR polyneuropath∗ OR cidp OR Guillain-Barre’s syndrome OR demyelinating disease∗ OR “polyradiculoneuropathy” OR “Miller Fisher’s syndrome∗”)

### 2.2 Data analysis

The data were exported to Microsoft Excel 2016. To obtain the bibliometric indicators, the SciVal software was used. The data were described through percentages in descriptive tables. Finally, some bibliometric indicators such as CiteScore, FWCI, SCimago Journal Rank, and h-index were used.

### 2.3 Ethics

None to declare.

## 3 Results

A total of 959 documents related to GBS and COVID-19 were collected in Medicine. The subcategories with the highest percentage were Neurology (481, 50.2%), General Medicine (134, 14.0%) and Infectious Diseases (93, 9.7%).

### 3.1 Top-10 authors on GBS and COVID-19

Josef Finsterer was the author with the highest average of publications (45 manuscripts), while Benedict Michael had the highest impact with 249.6 citations per paper in his 5 publications. Fúlvio Scorza and Ana Fiorini were the second and third most productive authors, respectively. Three of the ten authors with the highest academic production were Brazilian (Table 1).

**Table 1:** Top-10 authors on Guillain-Barré Syndrome and COVID-19.

Rank	Author	Documents n (%)	Total citation	Citations per document	h-index	FWCI	Country
1	Finsterer, Josef	45 (4.7)	109	2.4	34	2.2	
2	Scorza, Fúlvio Alexandre	24 (2.5)	85	3.5	36	3.7	
3	Fiorini, Ana Claudia	10 (1.0)	26	2.6	9	4.3	
4	Scorza, Carla Alessandra	8 (0.8)	7	0.9	19	0.8	
5	Benito-Léon, Julían	7 (0.7)	432	61.7	55	15.0	
6	Ghosh, Ritwik	6 (0.6)	77	12.8	9	5.0	
7	Nobile-Orazio, Eduardo	6 (0.6)	99	16.5	48	10.2	
8	Gigli, Gian Luigi	6 (0.6)	75	12.5	37	12.0	
9	Michael, Benedict Daniel	5 (0.6)	1248	249.6	22	37.8	
10	Benedetti, Luana	5 (0.6)	69	13.8	26	11.1	

FWCI: Field-weighted citation impact.

### 3.2 Top-10 institutions on GBS and COVID-19

The 10 institutions with the highest average of articles are shown in Table 2. The Universidade Federal de São Paulo (Brazil) was the university with the highest scientific production (29) while King’s College London had the highest impact (90.5 citations per manuscript). Harvard University (USA) and University College London (UK) were the second and third college with the highest academic production, respectively.

**Table 2:** Top-10 institutions on Guillain-Barré Syndrome and COVID-19.

Institution (country)	Documents n (%)	Total citation	Authors	Citations per document	FWCI
Universidade Federal de São Paulo (Brazil)	29 (3.0)	148	15	5.1	3.5
Harvard University (USA)	25 (2.6)	427	58	17.1	7.6
University College London (UK)	23 (2.4)	1929	79	83.9	21.6
Institut national de la santé et de la recherche médicale (France)	18 (1.9)	353	62	19.6	6.0
Johns Hopkins University (USA)	15 (1.6)	256	17	17.1	9.0
Complutense University (Spain)	13 (1.4)	545	28	41.9	12.9
University of Barcelona (SPain)	13 (1.4)	110	25	8.5	4.3
Centre National de la Recherche Scientifique (France)	13 (1.4)	331	62	25.5	7.4
King’s College London (UK)	13 (1.4)	1177	38	90.5	22.5
University of Milan (Italy)	12 (1.3)	158	37	13.2	7.6

FWCI: Field-weighted citation impact.

### 3.3 Production and impact on GBS and COVID-19

With respect to the journals, Journal of Neurology, Neurological Sciences and Frontiers in Neurology are the ones with the highest publications on the topic of study with 36, 32 and 22 manuscripts, respectively. However, the journal with the highest impact was Neurology with 54.2 citations per paper (Table 3).

**Table 3:** Top journals on Guillain-Barré syndrome and COVID-19.

Journals	Quartile	Article Processing Charges	Scimago Journal Rank	Documents	Citations	Citations per document	CiteScore 2020
Journal of Neurology	Q1	Yes	1.5	36	973	27.0	6.4
Neurological Sciences	Q2	Yes	0.8	32	661	20.7	4.0
Frontiers in Neurology	Q2	Yes	1.2	22	298	13.5	4.0
European Journal of Neurology	Q1	Yes	1.9	20	366	18.3	7.4
Neurology	Q1	Yes	2.9	19	1029	54.2	9.6
BMJ Case Reports	Q4	Yes	0.2	19	203	10.7	0.8
Journal of Medical Virology	Q2	Yes	0.8	17	171	10.1	11.6
Journal of Neuroimmunology	Q2	Yes	1.1	16	114	7.1	5.3
Annals of Indian Academy of Neurology	Q3	Yes	0.4	15	43	2.9	1.5
Muscle and Nerve	Q2	Yes	1.0	13	187	14.4	4.9

### 3.4 CiteScore quartil on GBS and COVID-19

Table 4 shows indicators of impact and production according to type of collaboration in Guillain-Barré Syndrome and COVID-19. Where an increasing distribution is observed with a higher percentage in Q1 level journals.

**Table 4:** Documents published according CiteScore Quartil 2020 on Guillain-Barré Syndrome and COVID-19.

CiteScore Quartile	2019	2020	2021	2022∗	Total
Q1	0	113	209	31	353
Q2	0	110	158	13	281
Q3	0	72	137	8	217
Q4	0	33	58	6	97
Total	0	328	562	58	948

∗ Documents published until 15 February.

### 3.5 Type of collaboration on GBS and COVID-19

Table 5 shows that most of the papers retrieved had only national collaboration (382 papers: 40.2%). The rest of the papers belong to the “single authorship” or “no collaboration” category (95 papers: 10.0%).

**Table 5:** Bibliometric indicators of production and impact according to type of collaboration on Guillain-Barré Syndrome and COVID-19.

Collaboration	%	Documents	Citations	Citations per document	FWCI
International	19.0	181	5282	29.2	9.0
Only national	40.2	382	5676	14.9	5.3
Only institutional	30.7	292	3486	11.9	3.5
Single authorship (no collaboration)	10.0	95	611	6.4	2.3

FWCI: Field-weighted citation impact.

## 4 Discussion

COVID-19 has gained importance in recent years, not only because of the pandemic it has caused but also because of its relationship with multiple chronic diseases, many of them without clear etiology, which in turn has generated new frontiers in knowledge and research opportunities. In this context, the purpose of our research was to evaluate the characteristics of publications on COVID-19 and GBS. This study was undertaken because of the growing body of literature that has attempted to describe a relationship between GBS in patients diagnosed with COVID-19. Because GBS is an acute inflammatory polyradiculoneuropathy and is a major cause of paralysis worldwide. In addition, it is considered a potential risk factor for exacerbating the picture of COVID-19 infection.

Our study found 959 publications between the period 2019–2022 with the area of neurology being the most frequent; in turn, Brazil was the country with the highest number of authors and most productive institutions.

Bibliometric studies are secondary studies that allow a global bibliometric analysis of the academic production of a specific topic. While no previous bibliometric study has performed an association between COVID-19 and GBS, Murat Kiraz [21] performed a global analysis, using the Web of Science (WoS) database, on COVID-19 publication in the areas of neurology and neurosurgery, finding that GBS was a very frequent research topic.

While Josef Finsterer was the author with the highest number of publications, Benedict Michael was the author with the highest impact by presenting the highest average of citations, despite reporting only 5 publications. This could be explained by the fact that these studies denote a high impact as they are published in high quality journals (Q1) in neurology; in addition, these studies also provide very important knowledge about neurological [22, 23, 24] or psychiatric [25] manifestations and complications in patients with COVID-19 as well as those who have received COVID-19 vaccine [26]. It should be noted that most of the most productive authors are Brazilian, which is consistent with a previous study that reported that Brazilian publications were among the most cited in the field of GBS and ZIKA virus infection [19]. In turn, another country that stands out among the authors is India, which in a previous study reported that between 1973 and 2012, it accounted for 2.92% of publications on GBS worldwide [18].

Regarding the most productive universities, although the Universidade Federal de São Paulo was the university with the highest average of publications, two British institutions, King’s College London and University College London, had the greatest impact worldwide. This could be explained by the fact that among their most cited publications on the subject are the first national surveillance study of neurological and psychiatric complications in COVID-19 [25] and reviews on the neurological spectrum associated with COVID-19 [23, 27, 28]. These results agree with previous bibliometric studies, King’s College London was reported as one of the universities with the highest average of citations in a study evaluating the 100 most cited articles on GBS up to 2016 using the WoS database [16]. In turn, University College London and the French and US institutions in our top 10 were reported in another study as the institutions with the highest number of COVID-19 manuscripts in neurology and neurosurgery during 2019–2020 [21]. All this would indicate that despite the pandemic these institutions have remained at the forefront in the field of GBS. Another aspect to note is that among the regions to which the most productive institutions belong, China is not found; this generates a peculiar situation since it is usually China that leads COVID-19 research [29]. This situation could be since some of the most productive regions in this study belong to Western Europe and North America, which are the regions with the highest prevalence of GBS worldwide [6], which would justify their greater concern to investigate this disease in the pandemic context.

Although the Journal of Neurology was the journal that reported the highest average of publications, Neurology was the journal that presented the greatest impact by having the highest number of citations worldwide. This journal has not only reported having a great relevance and citation in the GBS topic [16], but also presents a great impact at the COVID-19 level and the neurology area in general [21] and is considered as a high-quality journal in the GBS area, according to specialists' recommendations [17]. This would be explained by the most recent publications of the journal report many citations, one of them evaluating two cases of Miller Fisher Syndrome (variant of GBS) and its relationship with cranial polyneuritis in patients with COVID-19 [30]. Regarding the quartile of the journals, a growth of high-quality publications (Q1) has been observed between 2020 and 2021 and even up to February 2022 they were still presenting higher quantity with respect to the rest of quartiles. This could demonstrate that research regarding the relationship of COVID-19 and neurological diseases such as GBS is gaining importance and impact.

Regarding the type of collaboration presented by the studies, the majority had only national collaboration and this in turn was more than double that of international collaboration. However, in terms of impact, international collaboration was the most frequently reported. Similarly, a previous study reported that in the field of GBS and ZIKA, international collaboration is also important [19]. This would indicate that although there are few studies with an international scope on our topic, they are highly cited and that these characteristics are shared in several neurological areas.

Our study has some limitations. First, we only used the information provided by the Scopus database, so there could be other studies published in other databases, so this should be considered before generalizing. Another limitation would be that our study only analyzed journal documents and did not include gray literature such as government reports or technical reports.

## 5 Conclusion

To sum up, academic publications on COVID-19 and Guillan-Barre Syndrome have been increasing, especially in first quartile journals, which would indicate high quality. In addition, international collaboration reported higher impact during the pandemic and several of the countries with the highest production of GBS were those where GBS reported the highest number of cases.

## Declarations

### Author contribution statement

Carlos Quispe-Vicuña, Frank Mayta-Tovalino, Miguel Cabanillas-Lazo, Maria Eugenia Guerrero, Franco Mauricio: Conceived and designed the experiments; Performed the experiments; Analyzed and interpreted the data; Contributed reagents, materials, analysis tools or data; Wrote the paper.

John Barja-Ore; Contributed reagents, materials, analysis tools or data.

### Funding statement

This research did not receive any specific grant from funding agencies in the public, commercial, or not-for-profit sectors.

### Data availability statement

Data will be made available on request.

### Declaration of interest’s statement

The authors declare no conflict of interest.

### Additional information

No additional information is available for this paper.
